# Subdural hematoma caused by rupture of a posterior cerebral artery aneurysm

**DOI:** 10.17712/nsj.2016.4.20160388

**Published:** 2016-10

**Authors:** Mahmood D. Al-Mendalawi

**Affiliations:** *Department of Pediatrics, Al-Kindy College of Medicine, Baghdad University, Baghdad, Iraq*

*To the Editor*

We have read with interest the case report by Feng et al^1^ on the subdural hematoma (SDH) caused by rupture of a posterior cerebral artery (PCA) aneurysm. It is noteworthy that the etiology of intracranial aneurysm formation and rupture remains mostly unknown, but multiple genetic susceptibilities in conjunction with the environmental factors are considered to act together in the etiology.^2^ An acute SDH is a rare complication of aneurysmal subarachnoid hemorrhage (SAH) and it is associated with a poor clinical condition on admission and a poor outcome. Increasing age, sentinel headache, intracerebral hemorrhage, and aneurysms at the posterior communicating artery are independent risk factors for an acute SDH. However, patients with a basilar or vertebral aneurysm have a low risk of an acute SDH.^3^ The available data pointed out that cerebrovascular complications of human immunodeficiency virus (HIV) infection/ AIDS were reported as high as 34% in post mortem series. Most HIV infected patients suffered from ischemic strokes but there are a number of reports of patients presenting with SAH related specifically to aneurysm rupture.^4^ In a recently published literature review, it was observed that the relationship between HIV infection and the formation of aneurysms appears to be real.^5^ Though the Chinese national HIV infection prevalence is still low (0.0598%) and new HIV infections have been contained at a low level (50,000-100,000 each year),^6^ HIV infection is still considered an important health threat. I presume that HIV infection- associated PCA aneurysm ought to be considered and its rupture had contributed to the development of SDH in the case in question. Hence, CD4 count and viral overload estimations were solicited to be contemplated.

## Reply from the Author

We appreciate the insightful comments of Prof. Al-Mendalawi on our article.^1^ Prof. Mahmood Al-Mendalawi put forward a valuable point that HIV infection-associated aneurysms ought to be considered in cerebral artery aneurysms. We thoroughly agree with him that infection related examinations should be performed in cerebral aneurysms and their rupture caused intracranial hemorrhage.

Infectious intracranial aneurysms (IIA) are rare, which account for 0.7-5.4% of all intracranial aneurysms.^7^ Various infectious pathogens can cause cerebral aneurysms, whose rupture may lead to intracranial hemorrhage, by injuring the vascular wall.^8^ The most common organism linked to infectious intracranial aneurysms (IIA) is bacteria,^9^ and we have ever reported a bacterial endocarditis associated distal cerebral aneurysm of adolescent.^10^ Also, HIV, which can directly invade the central nervous system (CNS) in a short time after systemic infection, was reported to cause IIAs.^11^ Though it is rare, parasites infection associated IIAs were reported from time to time.^8^ For instance, we have reported cerebral aneurysms associated with cerebral paragonimiasis.^12^

Posterior cerebral artery aneurysms may be associated with vascular malformation, infection, head trauma and so on.^13^ In our case, the total white blood cell count of the patient was normal. And he had no history of blood transfusion or relevant history of HIV infection. In addition, the HIV antibody and HIV p24 antigen enzyme-linked immunosorbent assay (ELISA) tests were negative. And the ELISA tests for multiple parasites performed on his serum were also negative. Based on these data, we did not consider infection-associated PCA aneurysm in this case. Because of the lack of a known origin, we finally considered it was an idiopathic dissecting aneurysm.

*Zhou Feng, Zhi Chen*

*Department of Neurosurgery, Southwest Hospital, Third Military Medical University Chongqing, The People’s Republic of China*
